# Mdb1, a Fission Yeast Homolog of Human MDC1, Modulates DNA Damage Response and Mitotic Spindle Function

**DOI:** 10.1371/journal.pone.0097028

**Published:** 2014-05-07

**Authors:** Yi Wei, Hai-Tao Wang, Yonggong Zhai, Paul Russell, Li-Lin Du

**Affiliations:** 1 College of Life Sciences, Beijing Normal University, Beijing, China; 2 National Institute of Biological Sciences, Beijing, China; 3 Department of Cell and Molecular Biology, The Scripps Research Institute, La Jolla, California, United States of America; Cancer Research UK London Research Institute, United Kingdom

## Abstract

During eukaryotic DNA damage response (DDR), one of the earliest events is the phosphorylation of the C-terminal SQ motif of histone H2AX (H2A in yeasts). In human cells, phosphorylated H2AX (γH2AX) is recognized by MDC1, which serves as a binding platform for the accumulation of a myriad of DDR factors on chromatin regions surrounding DNA lesions. Despite its important role in DDR, no homolog of MDC1 outside of metazoans has been described. Here, we report the characterization of Mdb1, a protein from the fission yeast *Schizosaccharomyces pombe*, which shares significant sequence homology with human MDC1 in their C-terminal tandem BRCT (tBRCT) domains. We show that in vitro, recombinant Mdb1 protein binds a phosphorylated H2A (γH2A) peptide, and the phospho-specific binding requires two conserved phospho-binding residues in the tBRCT domain of Mdb1. In vivo, Mdb1 forms nuclear foci at DNA double strand breaks (DSBs) induced by the HO endonuclease and ionizing radiation (IR). IR-induced Mdb1 focus formation depends on γH2A and the phospho-binding residues of Mdb1. Deleting the *mdb1* gene does not overtly affect DNA damage sensitivity in a wild type background, but alters the DNA damage sensitivity of cells lacking another γH2A binder Crb2. Overexpression of Mdb1 causes severe DNA damage sensitivity in a manner that requires the interaction between Mdb1 and γH2A. During mitosis, Mdb1 localizes to spindles and concentrates at spindle midzones at late mitosis. The spindle midzone localization of Mdb1 requires its phospho-binding residues, but is independent of γH2A. Loss of Mdb1 or mutating its phospho-binding residues makes cells more resistant to the microtubule depolymerizing drug thiabendazole. We propose that Mdb1 performs dual roles in DDR and mitotic spindle regulation.

## Introduction

In eukaryotic cells, DNA lesions occur in the context of chromatin, and thus it is not surprising that many types of chromatin modifications have been implicated in DDR [1]–[5]. One of the first discovered DNA damage-associated chromatin modifications is the phosphorylation of the C-terminal SQ motif in mammalian histone H2AX, termed γH2AX [6], [7]. In yeasts, the SQ-motif-containing homologs of mammalian H2AX are the major histone H2A proteins, and they also undergo DNA damage-induced phosphorylation, termed γH2A [8]–[10]. γH2AX and γH2A are generated by phosphatidylinositol 3-kinase-related protein kinases including mammalian ATM, ATR, and DNA-PK, budding yeast Mec1 and Tel1, and fission yeast Rad3 and Tel1. Upon DNA damage, γH2AX or γH2A is induced within minutes and is among the earliest DDR events. The most dramatic phenotype caused by the loss of γH2AX or γH2A is the abolishment of ionizing radiation induced foci (IRIF) formed by many DDR factors [11]. Therefore, the main role of γH2AX and γH2A is believed to be a recruitment platform for the accumulation of DDR factors on chromatin surrounding DNA lesions.

Among the mammalian DDR proteins that rely on γH2AX for IRIF formation, MDC1 is the main direct binder of γH2AX [12], [13], and in turn is responsible for recruiting most other proteins that form IRIF in a γH2AX-dependent manner [14], [15]. The key structural feature of MDC1 that allows it to recognize γH2AX is its C-terminal tBRCT domain [12], [16], which has a single phosphopeptide binding pocket that specifically accommodates not only the phosphate moiety of pSer, but also the pSer +2 residue and the pSer +3 carboxylate in γH2AX.

In fission yeast, there are two major histone H2A proteins, H2A.1 and H2A.2, encoded by the genes *hta1* and *hta2*, respectively. When the SQ motifs in both H2A proteins are mutated, the resultant strain, *hta1-S129A hta2-S128A*, referred to as *htaAQ*, is sensitive to a wide range of genotoxins, and is partially defective in checkpoint maintenance [10]. The first identified γH2A binder in fission yeast is the DNA damage checkpoint mediator Crb2 [10], [17], [18], which requires not only γH2A, but also H4K20 methylation (H4K20me) for its IRIF formation [19], [20]. The function of Crb2 is not completely dependent on these histone modifications, because in the absence of γH2A or H4K20me, it can still be recruited to DNA lesions, albeit less efficiently, through a histone modification-independent pathway that relies on interactions with Rad4/Cut5, a homolog of mammalian TopBP1 [20], [21]. During S phase, another γH2A binder in fission yeast, Brc1, contributes to the repair of damaged replication forks and the maintenance of pericentromeric heterochromatin [22]–[24]. Crb2 and Brc1 are evolutionarily related to mammalian 53BP1 and PTIP, respectively. It has been unclear whether MDC1-like proteins exist in fission yeast and other non-metazoan eukaryotes.

Here we present our analysis of a fission yeast protein sharing significant sequence homology with human MDC1. It is encoded by the previously uncharacterized gene SPACUNK4.14. We named this protein Mdb1, for *m*idzone- and *D*NA-*b*reak-localizing protein. Our analysis demonstrates that Mdb1 is a direct γH2A binder, and influences both DDR and mitotic spindle function.

## Results

### Mdb1 is a Sequence Homolog of Human MDC1

**Figure 1:**
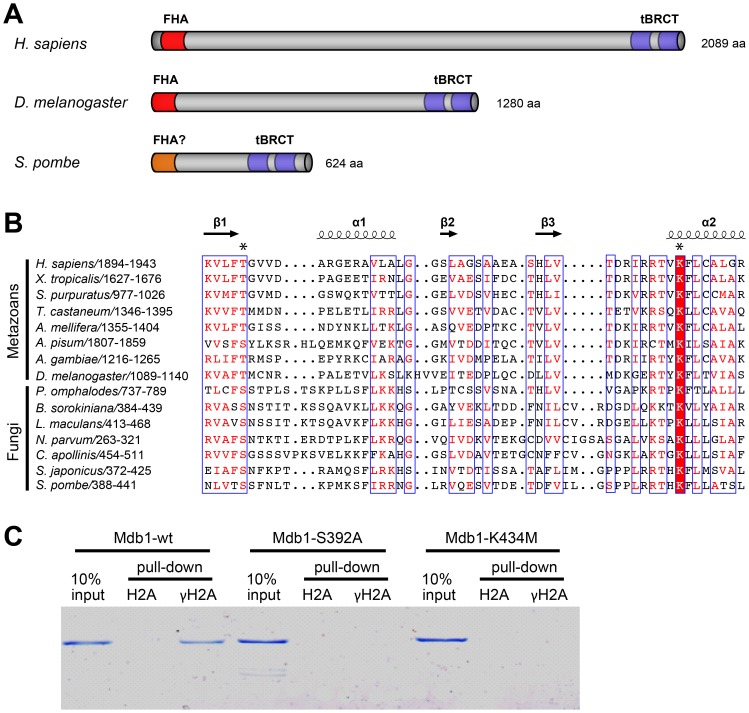
Mdb1 is a MDC1 homolog and binds γH2A directly. (A) A schematic showing the domain organizations of human MDC1 (accession NP_055456), *Drosophila* ortholog of human MDC1 (accession NP_523887) [44], and *S. pombe* Mdb1 (accession NP_593964). The FHA domains and tBRCT domains are highlighted in red and blue, respectively. The N-terminal region of Mdb1 (amino acids 1–89) is conserved in the fission yeast species and is predicted to be rich in beta strands by secondary structure predictions. We speculate that this region may adopt a FHA-like fold, despite lacking obvious sequence homology to any known FHA domains. (B) A multiple sequence alignment of the N-terminal portion of the tBRCT domain in metazoan proteins related to MDC1 and fungal proteins related to Mdb1. The alignment was generated by MAFFT-L-INS-i [45]. Secondary structural elements of human MDC1 (PDB 2ADO) were visualized together with the alignment using ESPript [46]. The two residues directly involved in γH2AX binding in human MDC1 (T1898 and K1936) are labeled with asterisks. For the alignment of the whole tBRCT domain and the accession numbers of the sequences, see Figure S1. (C) Mdb1 directly binds a γH2A peptide in a manner dependent on the conserved phospho-binding residues in the tBRCT domain. Wild-type and two mutant versions (S392A and K434M) of Mdb1 proteins were expressed in bacteria and purified using the His_6_ tag. Biotinylated peptides that correspond to the C terminus of H2A.1, either unmodified (H2A) or phosphorylated on Ser-129 (γH2A), were incubated with the recombinant Mdb1 proteins. Peptides and associated proteins were pulled down by streptavidin Dynabeads and eluted by boiling in SDS-PAGE loading buffer. The eluates and 10% inputs were analyzed by SDS-PAGE followed by Coomassie staining.

Mdb1 is a protein of 624 amino acids. BLASTP analysis and multiple sequence alignments indicate that its C-terminal region between amino acids 388–580 is strongly conserved (Figure 1A, 1B and Figure S1). In fungi, outside of the genus *Schizosaccharomyces*, apparent orthologs of Mdb1 can be found in a subset of species belonging to the subphylum Pezizomycotina, with BLASTP E-values ranging from 7e-21 for a protein from *Coniosporium apollinis* to 6e-11 for a protein from *Leptosphaeria maculans*. In metazoans, the closest homologs of Mdb1 are human MDC1 and proteins related to it. The conserved region in Mdb1 corresponds to the tBRCT domain of human MDC1 (amino acids 1894–2081) (Figure S1), indicating that Mdb1 also has a tBRCT domain. Within the tBRCT domain of human MDC1, T1898 and K1936, the two residues directly involved in binding the phosphate moiety of pSer in γH2AX [12], are conserved in Mdb1 and Mdb1-related fungal proteins. In Mdb1, the corresponding residues are S392 and K434 (Figure 1B). Furthermore, R1932 and R1933, the MDC1 residues involved in binding the pSer +2 residue and the pSer +3 carboxylate in γH2AX, respectively [12], are also conserved in Mdb1 (Figure 1B).

### Mdb1 Directly Binds γH2A in vitro

The resemblance of Mdb1 and MDC1 in their tBRCT domains prompted us to investigate whether Mdb1 is able to bind γH2A directly. As S392 and K434 were predicted to be the phospho-binding residues, we included in the analysis two mutant forms of Mdb1, in which serine 392 was substituted with alanine and lysine 434 was substituted with methionine, respectively. The wild type and the mutant versions of Mdb1 proteins, denoted as Mdb1-wt, Mdb1-S392A and Mdb1-K434M, were expressed and purified from *E. coli*. Two 13-amino-acid peptides corresponding to residues 120–132 of H2A.1 were synthesized, with serine 129 being unphosphorylated in one peptide (denoted as H2A) and phosphorylated in the other (denoted as γH2A). These two H2A peptides were used for in vitro Mdb1 pull-down assays. By Coomassie staining of proteins in a polyacrylamide gel, we found that Mdb1-wt can be pulled down by the γH2A peptide but not the H2A peptide (Figure 1C). Neither mutant form of Mdb1 was detected in any pull-down fractions (Figure 1C). These results indicate that S392 and K434 of Mdb1 mediate a direct and phosphorylation-dependent interaction between Mdb1 and γH2A.

### Mdb1 Relocalizes to DSBs through its Interaction with γH2A

**Figure 2:**
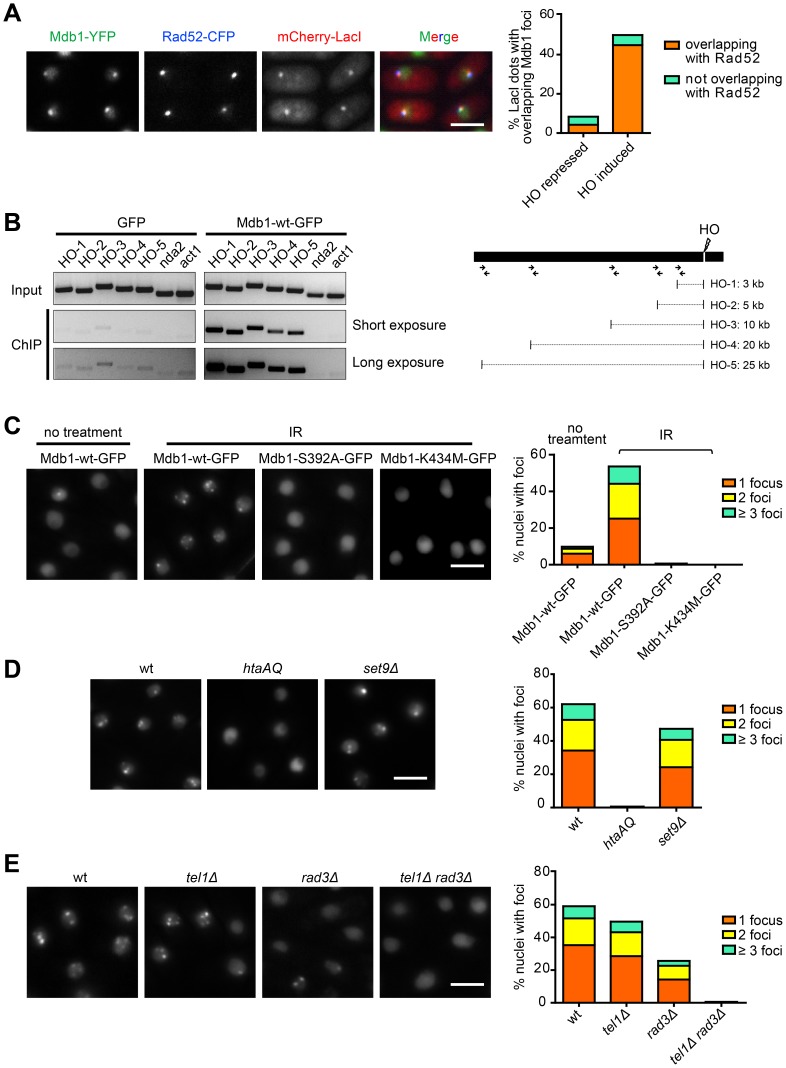
Mdb1 relocalizes to DSBs in a γH2A-dependent manner. (A) Mdb1-YFP forms nuclear foci at HO-induced DSBs. For HO endonuclease induction, cells were shifted to a thiamine-free medium for 12 h before imaging. The strain used was DY389. Bar, 5 µm. (B) Chromatin immunoprecipitation (ChIP) analysis using anti-GFP antibody showed that Mdb1-GFP, but not GFP alone, is recruited to chromatin regions adjacent to the HO-induced DSB. PCR primers used for amplifying DNA regions near the HO cleavage site are depicted in the schematic on the right. Primers amplifying the genes *nda2* and *act1* were used as negative controls. The strains used were DY16261 and DY16263. (C) The phospho-binding residues are required for Mdb1 IRIF formation. Mdb1 IRIF were imaged and quantified before and after exposure to 36 Gy of IR. To quantify the levels of foci-containing nuclei, about 200 nuclei were examined for each sample. Strains used were DY15603, DY15604, and DY15605. Bar, 5 µm. (D) Mdb1 IRIF formation requires γ-H2A but not H4K20me. Cells were treated with 36 Gy of IR and analyzed as in (B). Strains used were DY15603, DY15606, and DY15607. Bar, 5 µm. (E) Mdb1 IRIF formation depends on Tel1 and Rad3. Cells were treated with 36 Gy of IR and analyzed as in (B). Strains used were DY15631, DY15608, DY15609, and DY15610. Bar, 5 µm.

As the interaction between MDC1 and γH2AX promotes the DSB localization of MDC1 [12], we examined whether Mdb1 also relocalizes to DSBs through its interaction with γH2A. We firstly used a strain in which an HO-endonuclease-induced DSB is marked by the *lacO*/LacI system [25]. In this strain, a *lacO* repeat is inserted near the HO cleavage site, thus allowing the spatial location of the HO cleavage site to be marked by mCherry-LacI. In addition, CFP-tagged Rad52 (also known as Rad22), which forms nuclear foci at DSBs, is also expressed in this strain. When Mdb1-YFP was expressed in such a strain, upon HO induction, we observed that Mdb1-YFP formed distinct nuclear foci colocalizing with mCherry-LacI and CFP-Rad52 (Figure 2A), suggesting that Mdb1 is a DSB-localizing protein. ChIP-PCR analysis confirmed that Mdb1 was enriched at chromatin regions adjacent to the HO-induced DSB (Figure 2B).

We next examined whether Mdb1 forms IRIF. After treating cells with 36 Gy of IR, we observed that Mdb1-GFP rapidly formed IRIF (Figure 2C). We quantified the levels of Mdb1 nuclear foci and found that about 10% of nuclei in non-treated cells had spontaneous Mdb1-GFP foci, while after IR exposure, the percentage of foci-containing nuclei reached nearly 60% (Figure 2C). Thus, Mdb1 efficiently forms IRIF like human MDC1 and fission yeast Crb2.

We next tested whether the phospho-binding residues in Mdb1 are important for IRIF formation. We found that either S392A or K434M mutation abolished Mdb1 IRIF formation (Figure 2C), suggesting that DSB targeting of Mdb1 requires its phospho-binding capability.

To examine whether γH2A is the phosphorylated binding partner that mediates Mdb1 DSB localization, we used the *htaAQ* mutant strain in which the phosphorylation sites on both H2A.1 and H2A.2 have been mutated [10], [23]. No Mdb1 IRIF was detected in this strain (Figure 2D), indicating that γH2A is crucial for targeting Mdb1 to DSBs. On the other hand, Mdb1 IRIF formed normally in *set9Δ* mutant cells (Figure 2D), which lack H4K20me [19]. Thus, unlike Crb2, Mdb1 IRIF formation is not dependent on H4K20me. This is consistent with the fact that Mdb1 lacks an obvious methylation-binding domain, such as the tandem Tudor domain in Crb2 [26].

In fission yeast, γH2A is generated by both Rad3 and Tel1, the homologs of human ATR and ATM kinases, respectively [10]. Thus, we expected that Mdb1 IRIF formation should be dependent on these kinases. Indeed, we found that Mdb1 IRIF formation was abolished in *tel1Δ rad3Δ* cells, but not in either single mutant (Figure 2E). We did observe a moderate reduction of Mdb1 IRIF in *rad3Δ* cells, consistent with a more prominent role of Rad3 in IR-induced γH2A formation [10].

### *mdb1Δ* Alters the DNA Damage Sensitivity of *crb2Δ* in a γH2A-dependent Manner

**Figure 3:**
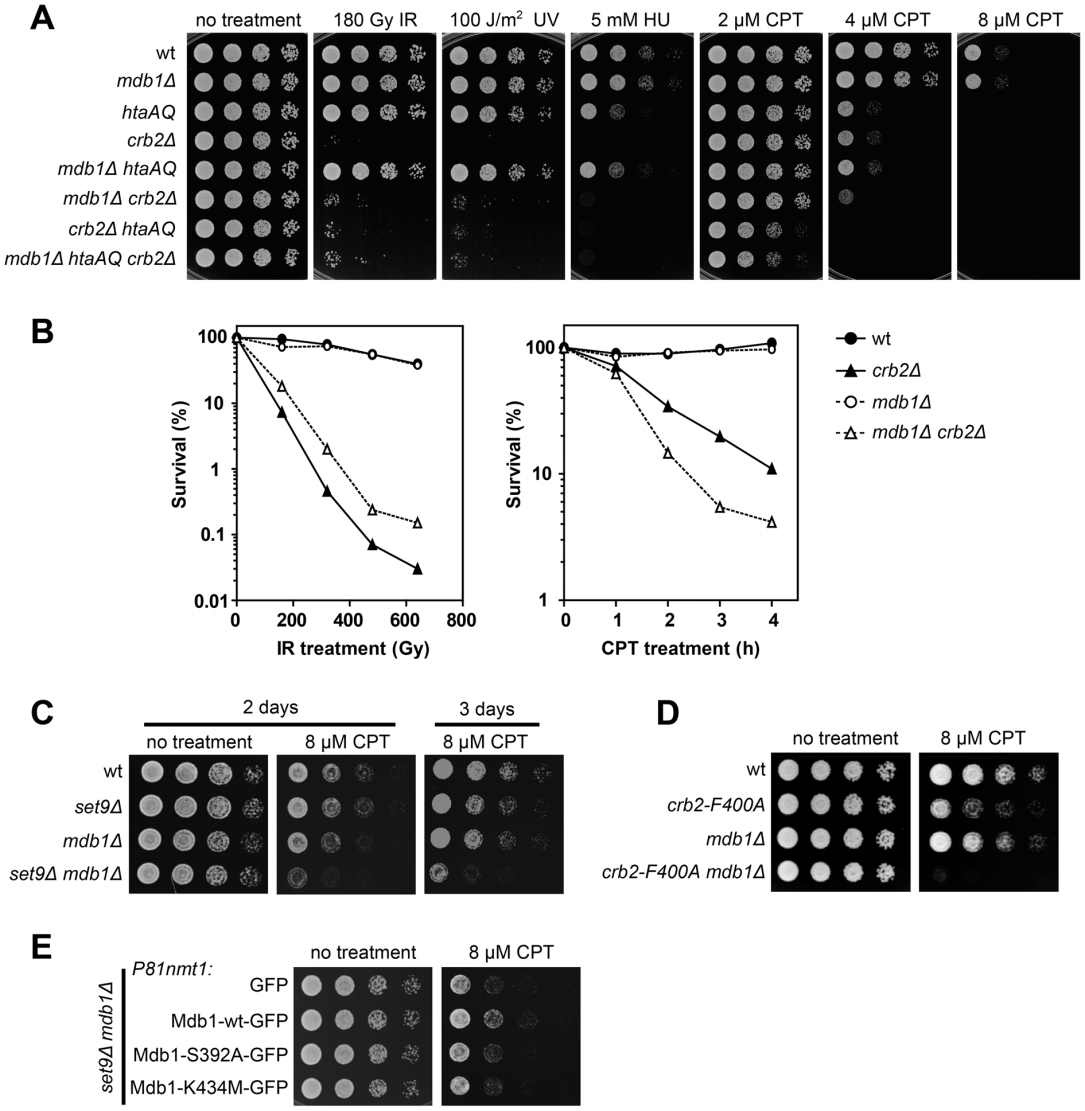
Loss of Mdb1 alters genotoxin sensitivity of *crb2* and *set9* mutants. (A) *mdb1Δ* alters the genotoxin sensitivity of *crb2Δ* in a manner similar to *htaAQ*. Strains used were LD327, LD1067, LD574, LD197, LD1068, LD1070, LD678, and LD1071. (B) Quantitative survival curve analysis showed that *mdb1Δ crb2Δ* double mutant is less sensitive to IR, but more sensitive to CPT than *crb2Δ* single mutant. For the CPT treatment, cells were exposed to 20 µM of CPT for the duration indicated. Strains used were LD327, LD1067, LD197, and LD1070. (C) *mdb1Δ set9Δ* double mutant is more sensitive to CPT than each single mutant. Strains used were LD259, LD723, LD964, and DY15611. (D) *mdb1Δ* enhances the CPT sensitivity of a Tudor domain mutation in *crb2*, *crb2F400A*. Strains used were LD260, LD744, LD1011, and DY16269. (E) The phospho-binding residues are required for rescuing the CPT sensitivity of *mdb1Δ set9Δ* double mutant. GFP, Mdb1-wt-GFP, Mdb1-S392A-GFP, and Mdb1-K434M-GFP were expressed under the control of the *P81nmt1* promoter. Cells were pregrown in a thiamine-free medium for 20 h before being spotted on CPT-containing thiamine-free plates. Strains used were DY15612, DY15607, DY15613, and DY15614.

To further investigate the physiological role of Mdb1, we generated an *mdb1Δ* strain and examined its DNA damage sensitivity (Figure 3A). No obvious sensitivity to IR, UV, hydroxyurea (HU), or camptothecin (CPT) was observed. We hypothesized that the lack of phenotype may be due to a redundancy with other γH2A binders, and thus examined the DNA damage sensitivity of the double mutant *mdb1Δ crb2Δ* (Figure 3A). Interestingly, *mdb1Δ crb2Δ* appeared to be less sensitive to IR and UV than *crb2Δ*, and more sensitive to CPT than *crb2Δ*. Quantitative survival curve analysis confirmed the results of the spot assay (Figure 3B). These effects of *mdb1Δ* on *crb2Δ* mimic those of *htaAQ* [10], [20], which suppressed the IR and UV sensitivity of *crb2Δ* to the same extents as *mdb1Δ*, and enhanced the CPT sensitivity of *crb2Δ* to a greater extent than *mdb1Δ* (Figure 3A). These results suggest that the interaction between Mdb1 and γH2A may underlie the stronger IR and UV sensitivity of *crb2Δ* compared to *crb2Δ htaAQ*, and partially account for the stronger CPT sensitivity of *crb2Δ htaAQ* compared to *crb2Δ*. Consistent with these interpretations, the triple mutant *mdb1Δ crb2Δ htaAQ* displayed the same phenotype as *crb2Δ htaAQ* (Figure 3A), indicating that the effects of *mdb1Δ* on *crb2Δ* require the presence of γH2A.

### *mdb1Δ* Enhances the CPT Sensitivity of *set9Δ* and *crb2-F400A*

The enhanced CPT sensitivity of *mdb1Δ crb2Δ* compared to *crb2Δ* suggests that Mdb1 and Crb2 may act redundantly during CPT response. To determine whether the histone modification-dependent Crb2 recruitment pathway contributes to this overlapping function, we combined *mdb1Δ* with *set9Δ*, which abolishes the histone modification-dependent Crb2 recruitment [20]. *set9Δ mdb1Δ* double mutant showed much stronger CPT sensitivity than the single mutants (Figure 3C), suggesting that the role of Mdb1 in CPT response may overlap with that of Crb2 molecules bound to modified histones. To more directly examine the role of histone-binding by Crb2, we used a Tudor domain mutation, *crb2-F400A*, which has been shown to disrupt IRIF formation by Crb2 [20]. Consistent with the *set9Δ mdb1Δ* double mutant data, the double mutant of *crb2-F400A mdb1Δ* was much more sensitive to CPT than either *crb2-F400A* or *mdb1Δ* (Figure 3D).

In a complementation analysis, we found that the CPT sensitivity of *mdb1Δ set9Δ* can be rescued by GFP-tagged Mdb1-wt, but not Mdb1-S392A or Mdb1-K434M, when these proteins were expressed from the weakest version of *nmt1* promoter, *P81nmt1* (Figure 3E), indicating that Mdb1 promotes CPT response through its interaction with γH2A.

### Mdb1 Overexpression Causes Sensitivity to Genotoxin Treatment

**Figure 4:**
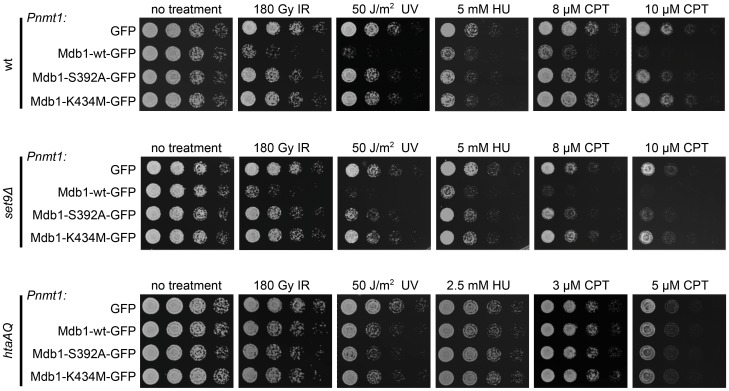
Overexpression of Mdb1 alters genotoxin sensitivity. Overexpression of wild-type but not phospho-binding mutants of Mdb1 results in genotoxin sensitivity in wild type and *set9Δ*, but not *htaAQ* strain background. Cells were pregrown in a thiamine-free medium for 20 h before being spotted on YES-based plates. Strains used were DY15615, DY15616, DY15617, DY15618, DY15619, DY15620, DY15621, DY15622, DY15623, DY15624, DY15625, and DY15626.

When performing the complementation analysis depicted in Figure 3E, we noticed that using the strong *Pnmt1* promoter to drive the expression of Mdb1-wt did not rescue, but rather exacerbated the CPT sensitivity of *mdb1Δ set9Δ* (our unpublished observation), suggesting that overexpression of Mdb1 can cause deleterious effects. To further examine Mdb1 overexpression-induced phenotype, we expressed GFP alone, Mdb1-wt-GFP, Mdb1-S392A-GFP, or Mdb1-K434M-GFP under the control of the *Pnmt1* promoter in the wild type background. As shown in Figure 4, we found that Mdb1 overexpression renders the cells sensitive to IR, UV, HU and CPT. Overexpression-induced sensitivities requires the two phospho-binding residues. Furthermore, when overexpression was carried out in histone modification mutants, heightened levels of DNA damage sensitivities were observed in *set9Δ* but not *htaAQ* mutant (Figure 4), indicating that overexpression of Mdb1 interferes with DNA damage response through its interaction with γH2A, and this interference does not require the histone modification-dependent recruitment of Crb2.

### Mdb1 Localizes to Mitotic Spindles Independently of γH2A

**Figure 5:**
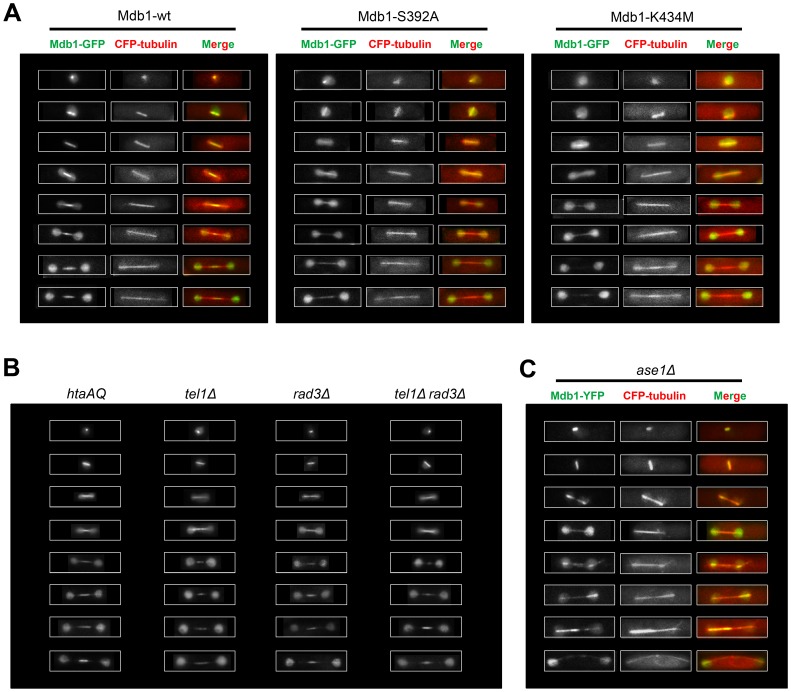
Mdb1 localizes to mitotic spindles. (A) Mdb1-GFP co-localizes with CFP-tubulin-labeled spindles and concentrates at the spindle midzone during late mitosis. The midzone accumulation of Mdb1 requires the phospho-binding residues in the tBRCT domain. Strains used were DY15627, DY15628, and DY15629. (B) The spindle localization of Mdb1 is independent of γH2A and Rad3/Tel1. Strains used were DY15606, DY15608, DY15609, and DY15610. (C) *ase1Δ* abolishes the midzone localization of Mdb1. Strain used was DY42.

While performing live cell imaging on GFP-tagged Mdb1, we noticed that it localizes to bar-shaped structures in a subset of cells of a log phase culture, and unlike the DSB foci, this type of localization was not enhanced by IR treatment. The shape of these structures suggests that they may correspond to mitotic spindles. Thus, we performed colocalization analysis using CFP–tubulin (Atb2) as a spindle marker. Indeed, the Mdb1-GFP labeled bar-shaped structures always overlapped with mitotic spindles marked by CFP–tubulin. Representative images of cells at different stages of mitosis are shown in Figure 5A. During early mitosis, when spindles are short, Mdb1 exhibits almost identical localization patterns as CFP–tubulin. During late mitosis, Mdb1 concentrates at the middle portion of the spindle, suggesting that Mdb1 becomes restricted to the midzone of the spindle.

Mutating the phospho-binding residues in the tBRCT domain abolished midzone localization of Mdb1 during late mitosis (Figure 5A). Mdb1-S392A could still be colocalized with CFP-tubulin during early mitosis, whereas Mdb1-K434M was hardly detectable on spindles even during early mitosis. Thus, the phospho-binding capability of Mdb1 is essential for its spindle midzone localization and may play a role in its localization to spindles during early mitosis. It has been shown for Crb2 that the two phospho-binding residues in the tBRCT domain act cooperatively and mutating one of them is not sufficient to completely abrogate phospho-binding [18]. Thus, it is possible that the spindle localization of Mdb1-S392A during early mitosis is due to residual phospho-binding.

We next tested whether γH2A or other Rad3/Tel1-mediated phosphorylation events are important for the mitotic localization of Mdb1 on spindles. We found that Mdb1 spindle localization was not affected in *htaAQ*, *tel1Δ*, *rad3Δ*, and *tel1Δ rad3Δ* mutant cells (Figure 5B). Thus, one or more proteins phosphorylated by kinase(s) other than Rad3 and Tel1 may bind to the Mdb1-tBRCT domain during mitosis and mediate the spindle localization of Mdb1.

### Ase1 is Important for the Spindle Midzone Localization of Mdb1

The spindle midzone is formed by antiparallel microtubule arrays. Ase1 bundles antiparallel microtubules and is a key player in spindle midzone assembly [27]–[29]. It has been reported that nearly all midzone components require Ase1 for proper localization [30], [31]. We analyzed the localization of Mdb1 in Ase1-deficient cells. As shown in Figure 5C, in *ase1Δ* cells, the accumulation of Mdb1 on the short spindles during early mitosis is not affected, but Mdb1 no longer concentrates at the middle region of the long spindles during late mitosis. Thus, like other midzone components, Mdb1 also requires Ase1 for the midzone localization.

### *mdb1Δ* Cells are Resistant to a Microtubule-destabilizing Drug

**Figure 6:**
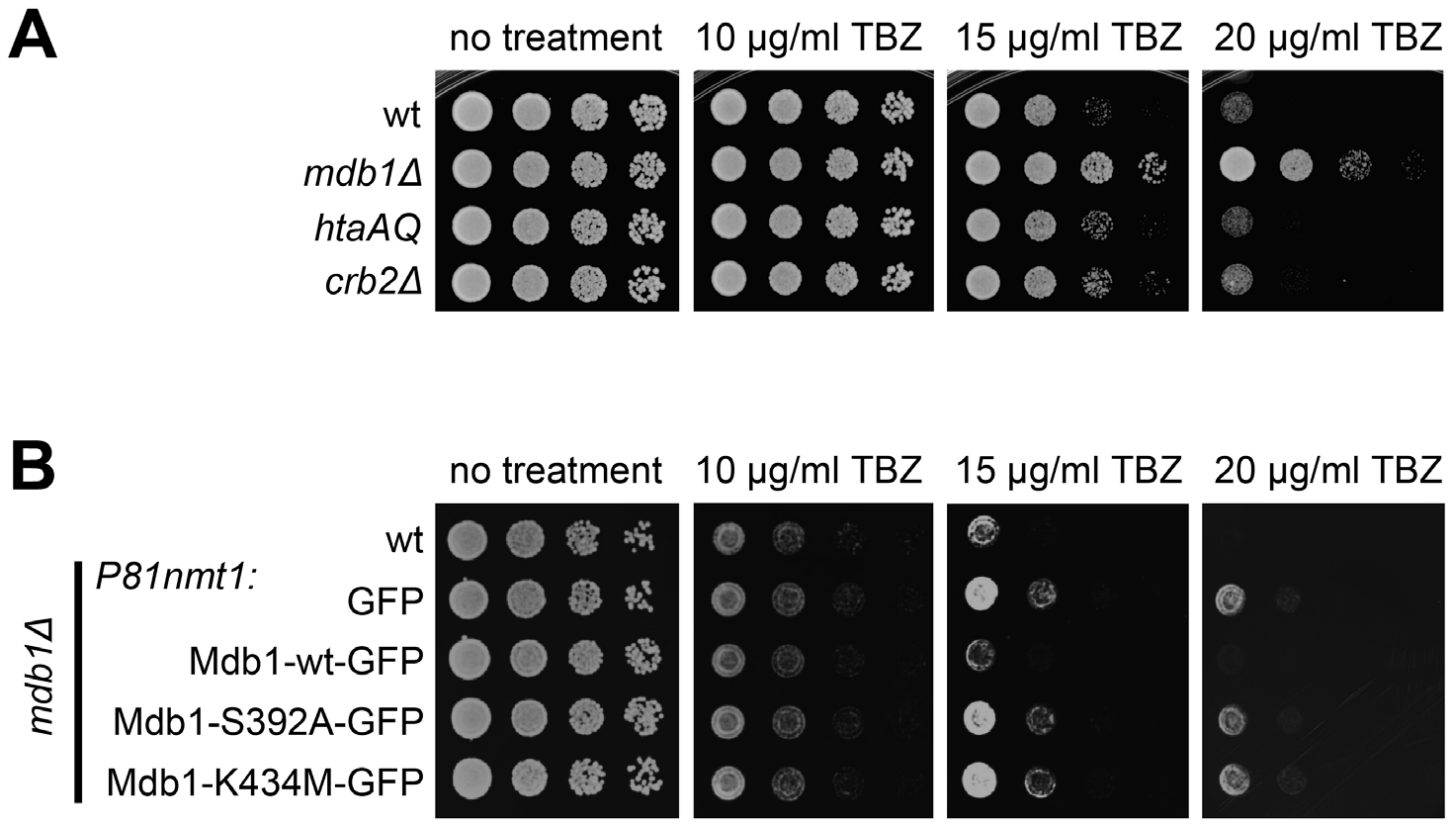
*mdb1* *Δ* causes resistance to the microtubule-depolymerizing drug TBZ. (A) *mdb1Δ*, but not *htaAQ* and *crb2Δ* mutants, was strongly resistant to TBZ. Cells were spotted on YES-based plates. Strains used were LD327, LD1067, LD574, and LD197. (B) The phospho-binding residues of Mdb1 are required for reversing the TBZ-resistant phenotype of *mdb1Δ*. Cells were pregrown in a thiamine-free medium for 20 h before being spotted on TBZ-containing thiamine-free plates. Strains used were LD327, DY15630, DY15603, DY15604, and DY15605.

Compared to the wild type, mutants defective in spindle midzone components often have altered sensitivity to microtubule-destabilizing drugs. For example, *ase1Δ* cells are more sensitive to methyl 2-benzimidazolecarbamate (MBC) [27], [28], whereas mutants defective in the kinesin-8 proteins Klp5 and Klp6 are more resistant to thiabendazole (TBZ) [32], [33]. To investigate the physiological relevance of Mdb1 spindle midzone localization, we examined the sensitivity of *mdb1Δ* cells to TBZ. As shown in Figure 6A, *mdb1Δ* cells were much more resistant to TBZ than wild type cells, whereas *htaAQ* and *crb2Δ* mutations did not significantly alter TBZ sensitivity, indicating that Mdb1 plays a role in spindle regulation independently of γH2A. Furthermore, we found that the TBZ-resistant phenotype of *mdb1Δ* can be reversed by *P81nmt1*-driven expression of Mdb1-wt-GFP but not Mdb1-S392A-GFP or Mdb1-K434M-GFP (Figure 6B). Thus, the phospho-binding residues in the tBRCT domain are not only required for spindle midzone localization of Mdb1, but are also important for conferring a normal level of TBZ sensitivity.

## Discussion

In this study, we show that Mdb1 is related to human MDC1 in the amino acid sequence, shares with MDC1 the biochemical property of directly binding γH2A(X) using the phospho-binding residues in the tBRCT domain, and behaves like MDC1 in forming γH2A-dependent IRIF. Furthermore, Mdb1 acts with other γH2A-binders in regulating DDR, although this role is largely masked by redundancy in the wild type background. Unexpectedly, our analyses also demonstrate that Mdb1 has a γH2A-independent function during mitosis.

### The Evolution of MDC1 Family Proteins

The identification of Mdb1 as a sequence and functional homolog of human MDC1 suggests that MDC1-like proteins existed in the ancestors of Opisthokonta supergroup (including Metazoa and Fungi) [34], but have since been lost in many fungal lineages including the budding yeast. The evolutionary origin of this protein family may go back even further, because in some non-Opisthokonta species, there are apparent sequence homologs of MDC1/Mdb1, for example, a protein from *Polysphondylium pallidum* (accession EFA74881) and a protein from *Dictyostelium discoideum* (accession EAL70982). Interestingly, like metazoan MDC1 proteins, these two amoebozoan proteins contain not only a C-terminal tBRCT domain, but also a N-terminal FHA domain, suggesting that such a domain combination may be a conserved feature of this protein family.

No obvious FHA domain can be detected in Mdb1, but its N-terminal 90 amino acids, about the size of a FHA domain, is well conserved among the species in the genus *Schizosaccharomyces*. We suspect that this region of Mdb1 may structurally correspond to the FHA domain in metazoan MDC1 proteins, but has diverged too much at the sequence level to be recognized by domain search tools.

### The Role of Mdb1 in DNA Damage Response

The loss of γH2A in fission yeast results in sensitivities to a wide varieties of genotoxins [10]. Disrupting either the binding between γH2A and Crb2, or the binding between γH2A and Brc1, using point mutations in Crb2 or Brc1, also leads to mild but detectable genotoxin sensitivity [18], [22]. In contrast, we found that the loss of Mdb1 causes no detectable genotoxin sensitivity. Thus, among the known downstream effectors of γH2A in fission yeast, Crb2 and Brc1 each appear to play non-replaceable γH2A-dependent roles, whereas Mdb1 mainly contributes in a way that is redundant with the other effectors.

The functional overlap between Mdb1 and Crb2 is evident in CPT response, where γH2A-bound Mdb1 and histone modification-recruited Crb2 redundantly promote the survival in the presence of CPT. Further analysis will be needed to elucidate the exact role shared by these two γH2A binders.

In IR and UV responses, our observation that *mdb1Δ* suppresses the phenotype of *crb2Δ* provides a new insight into the negative role of γH2A in the survival of *crb2Δ* cells against IR and UV [10], [20]. Our data suggest that γH2A-bound Mdb1 needs to be counteracted by Crb2 to allow proper repair of DNA lesions generated by IR and UV. Thus, depending on the circumstances, downstream effectors of γH2A may act either synergistically, or antagonistically. The negative impact of Mdb1 on IR and UV response in *crb2Δ* cells is likely related to the DNA damage sensitivity caused by Mdb1 overexpression. As the Mdb1 overexpression-induced phenotypes are independent of histone-dependent recruitment of Crb2 (Figure 4), we propose that the deleterious effect of Mdb1 overexpression is mainly exerted through hyper-activating its downstream effectors rather than through competing with Crb2 for γH2A binding. Consistent with this model, we found that overexpression of Mdb1 does not significantly affect the ability of Crb2 to form IRIF (Figure S2).

Both Crb2 and Brc1 have γH2A-independent DDR functions [20], [22]. However, all DDR-related readouts of Mdb1 functions, including IRIF formation, effects of *mdb1Δ* on *crb2Δ* and *set9Δ*, and Mdb1 overexpression-induced genotoxin sensitivity, are dependent on γH2A. Thus, Mdb1 appears to act strictly downstream of γH2A in DDR.

### The Role of Mdb1 in Spindle Regulation

The γH2A-independent spindle localization of Mdb1 suggests that it may play a role unrelated to DDR during mitosis. This model is supported by the TBZ-resistant phenotype of *mdb1Δ*. The spindle midzone localization of Mdb1 relies on the phospho-binding residues in the tBRCT domain. It has been shown that the tBRCT domain of human MDC1 binds phosphoproteins other than γH2AX [35], [36]. Thus, we hypothesize that a phosphorylated spindle component may be responsible for recruiting Mdb1 to the spindle midzones.

To our knowledge, the only known fission yeast mutants strongly resistant to TBZ are loss-of-function mutants of the Klp5-Klp6 complex [32], [33], or mutants perturbing the C-terminal region of Dam1, a component of the Dam1/DASH complex [37]–[39]. Like Mdb1, the Klp5-Klp6 complex also accumulates at spindle midzone [32], [33]. Thus, it is tempting to speculate that Mdb1 may be functionally related to Klp5-Klp6. The midzone localizations of Mdb1 and Klp5-Klp6 do not depend on each other (our unpublished observations). Therefore, even if they act together, they cannot be simply placed into a linear recruitment pathway.

In one quantitative proteomic study, Mdb1 was found among the proteins most down-regulated as cells progressed from G2 to G1/S [40]. In another large scale analysis, Mdb1 was shown to be a mitotic substrate of the Aurora-related kinase Ark1 [41]. It is likely that the mitotic function of Mdb1 is controlled by post-translational events.

## Materials and Methods

### Fission Yeast Strains and Media

The fission yeast strains used in this study are listed in Table S1. Genetic methods for strain construction and composition of media are as described [42]. For the construction of plasmids expressing Mdb1-GFP, the Mdb1 coding sequence was amplified by PCR and inserted into modified pDUAL vectors [43], which contain one of the three versions of thiamine-repressible *nmt1* promoter (*Pnmt1*, *P41nmt1*, or *P81nmt1*) and the sequence encoding GFP. The plasmids were linearized with NotI and integrated at the *leu1* locus, or linearized with MluI and integrated at the *ars1* locus. The point mutations in Mdb1 were generated by overlap PCR. For the strains containing CFP-tubulin (CFP-Atb2), pREP81-CFP-atb2 (a gift from Yoshinori Watanabe) was linearized with MluI and integrated at the *ars1* locus. The Mdb1-YFP strain was constructed by integrating at the *mdb1* locus a plasmid containing the sequence encoding a C-terminal fragment of the Mdb1 fused with YFP, so that full-length Mdb1 fused with YFP is expressed from the endogenous promoter.

### Mdb1 Purification and H2A Peptide Pull Down

His_6_-tagged Mdb1 proteins were expressed in *E. coli* strain BL21 and purified using Ni-NTA beads (Qiagen) in accordance with manufacturer’s instructions. Two peptides that correspond to the 13 C-terminal residues of H2A.1 (amino acids 120–132) were synthesized with an N-terminal biotin, with one peptide having an unmodified serine, and the other having a phospho-serine at position 129. About 5–10 µg of purified Mdb1 was incubated with 2 µg of peptide in 200 µl of peptide binding buffer (50 mM Tris-HCl, pH 7.5, 100 mM NaCl, 0.05% NP-40) at 4°C for 2 hours. Then 20 µl of pre-washed Dynabeads M-280 Streptavidin (Invitrogen) was added to pull down the peptide and peptide-associated protein. Beads were washed with peptide binding buffer and eluted with SDS-PAGE loading buffer.

### Live Cell Imaging

Light microscopy was performed using a DeltaVision PersonalDV system (Applied Precision) equipped with a CFP/YFP/mCherry filter set (Chroma 89006 set) and a Photometrics CoolSNAP HQ2 camera. Images were acquired with a 100×, 1.4-NA objective, and analyzed with the SoftWoRx software.

### ChIP Assay

ChIP assay was performed as described [20]. GFP-trap beads (Chromotek) were used for enriching chromatin bound by GFP-tagged Mdb1. PCR primers used for the ChIP assay are listed in Table S2.

### Genotoxin and TBZ Sensitivity Assays

For the spot assay, cultures were grown to log phase and serial 5-fold dilutions of cells were spotted onto control and genotoxin-containing plates. UV irradiation was applied using a CL-1000 UV crosslinker (UVP). For IR treatment, cells were irradiated with a Gammacell 1000 irradiator (dose rate 16 Gy/min) in microfuge tubes and then plated on YES plates. For the quantitative survival curve analysis, cells were irradiated by IR at the indicated dose, or exposed to CPT for the indicated duration, and then plated after appropriate dilution on YES plates to obtain colony counts. Colony counts were normalized against the colony counts of IR treatment of 0 Gy or CPT treatment at zero time point.

## Supporting Information

Figure S1 **Full alignment of the tBRCT domain sequences shown in** **Figure 1B** **.** The alignment was generated by MAFFT-L-INS-i [45]. Secondary structural elements of human MDC1 (PDB 2ADO) were visualized together with the sequence alignment using the ESPript web server (http://espript.ibcp.fr/) [46]. Genbank accession numbers are gi|86197957 (*Homo sapiens*), gi|512859699 (*Xenopus tropicalis*), gi|390363726 (*Strongylocentrotus purpuratus*), gi|270009477 (*Tribolium castaneum*), gi|328783997 (*Apis mellifera*), gi|328702829 (*Acyrthosiphon pisum*), gi|158294073 (*Anopheles gambiae*), gi|24655776 (*Drosophila melanogaster*), gi|549055123 (*Pyronema omphalodes*), gi|451845536 (*Bipolaris sorokiniana*), and gi|312218784 (*Leptosphaeria maculans*), gi|485920914 (*Neofusicoccum parvum*), gi|494826952 (*Coniosporium apollinis*), gi|530774456 (*Schizosaccharomyces japonicus*), and gi|380865396 (*Schizosaccharomyces pombe*).
(PDF)

Figure S2 **Crb2 IRIF formation remains largely normal when Mdb1 is overexpressed from the** ***Pnmt1*** **promoter.** Expressing Mdb1-GFP from the strong *Pnmt1* promoter did not obviously alter the level of Crb2 IRIF, compared to cells expressing Mdb1-GFP from the weak *P81nmt1* promoter. IRIF formed by mCherry-tagged Crb2(276–778) and Mdb1-GFP were imaged and quantified after exposure to 36 Gy of IR. Strains used were DY15912 and DY15602. Bar, 3 µm.
(PDF)

Table S1 **The strains used in this study.**
(PDF)

Table S2 **The PCR primers used in the ChIP assay.**
(PDF)
