# Supplementary figures and images for: Mdb1, a Fission Yeast Homolog of Human MDC1, Modulates DNA Damage Response and Mitotic Spindle Function
Source: PLoS One. 2014 May 7;9(5):e97028. doi: 10.1371/journal.pone.0097028 (PMC4013092; doi:10.1371/journal.pone.0097028)

# Wei et al. Figure S1

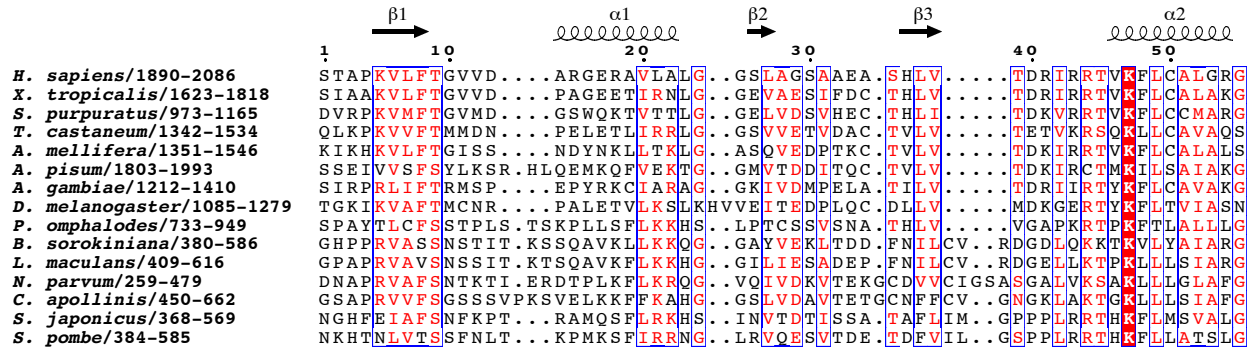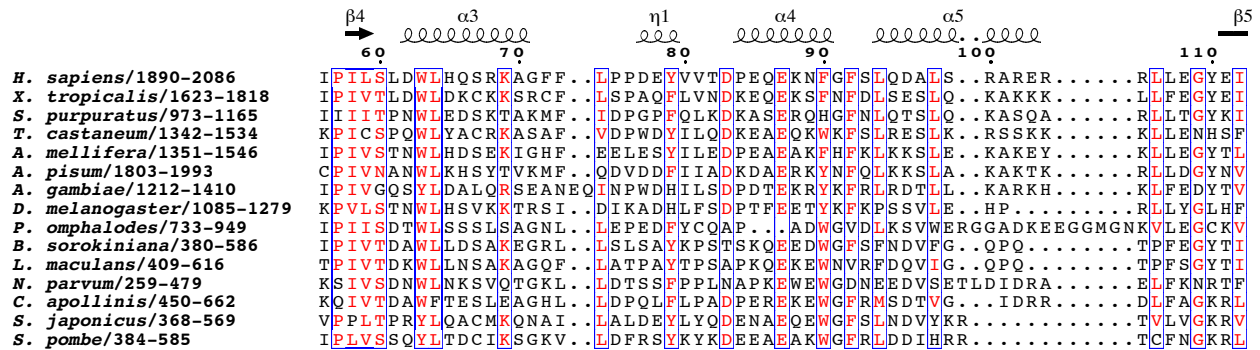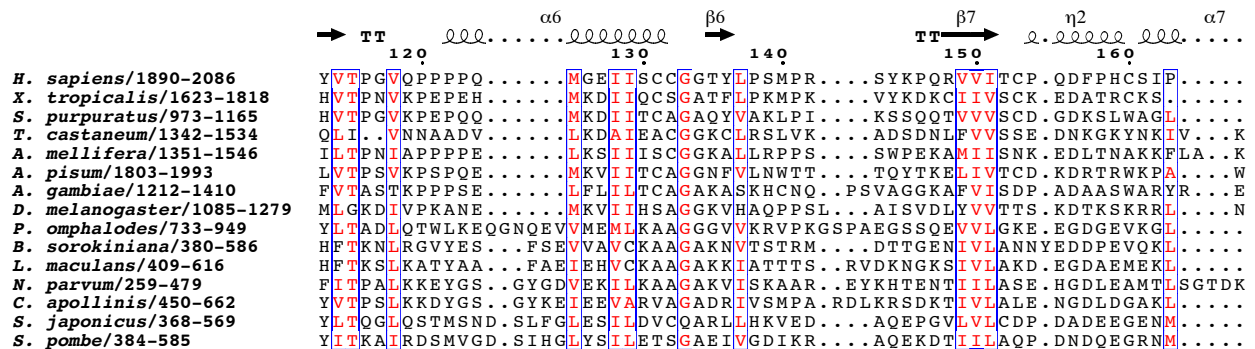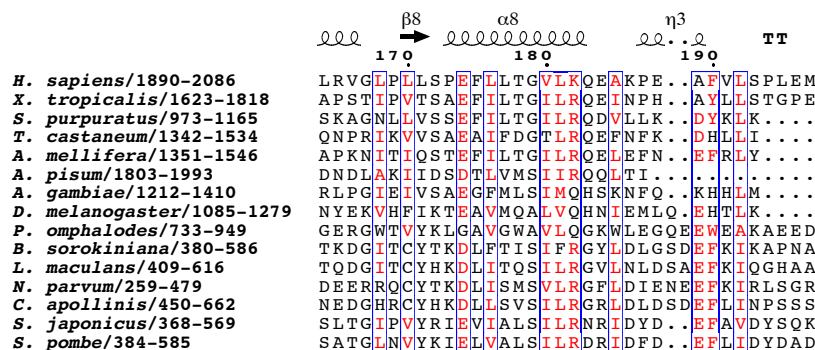

Supplement: Figure S1 — Full alignment of the tBRCT domain sequences shown in Figure 1B . The alignment was generated by MAFFT-L-INS-i [45]. Secondary structural elements of human MDC1 (PDB 2ADO) were visualized together with the sequence alignment using the ESPript web server (http://espript.ibcp.fr/) [46]. Genbank accession numbers are gi|86197957 (Homo sapiens), gi|512859699 (Xenopus tropicalis), gi|390363726 (Strongylocentrotus purpuratus), gi|270009477 (Tribolium castaneum), gi|328783997 (Apis mellifera), gi|328702829 (Acyrthosiphon pisum), gi|158294073 (Anopheles gambiae), gi|24655776 (Drosophila melanogaster), gi|549055123 (Pyronema omphalodes), gi|451845536 (Bipolaris sorokiniana), and gi|312218784 (Leptosphaeria maculans), gi|485920914 (Neofusicoccum parvum), gi|494826952 (Coniosporium apollinis), gi|530774456 (Schizosaccharomyces japonicus), and gi|380865396 (Schizosaccharomyces pombe). (PDF) [file pone.0097028.s001.pdf]

Wei et al. Figure S2

*Pnmt1*-driven Mdb1-GFP

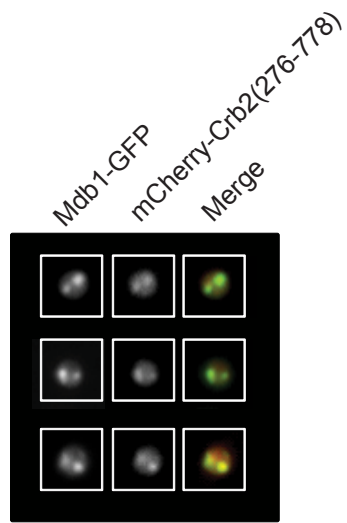

*Pnmt81*-driven Mdb1-GFP

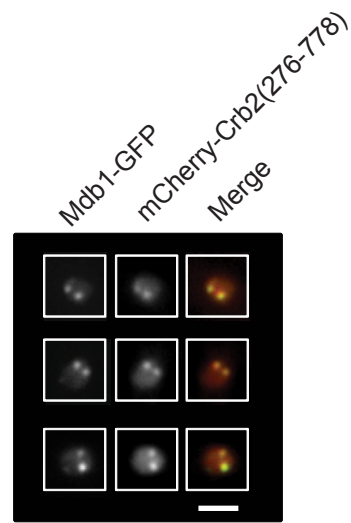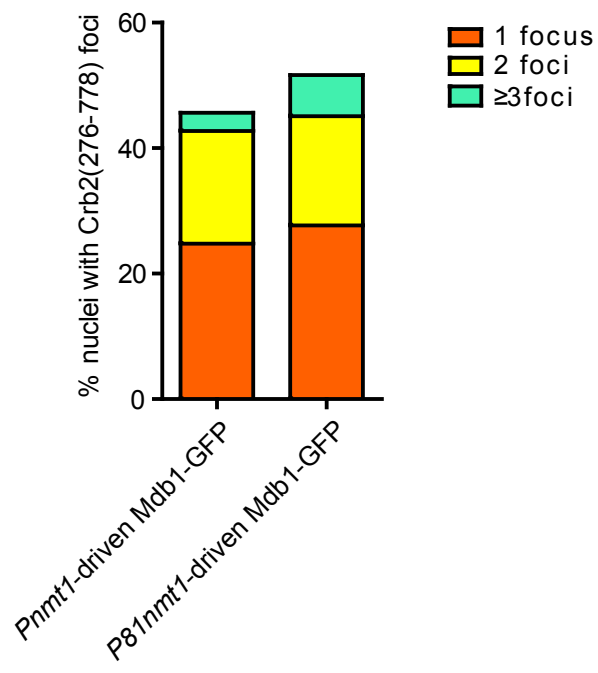

Supplement: Figure S2 — Crb2 IRIF formation remains largely normal when Mdb1 is overexpressed from the Pnmt1 promoter. Expressing Mdb1-GFP from the strong Pnmt1 promoter did not obviously alter the level of Crb2 IRIF, compared to cells expressing Mdb1-GFP from the weak P81nmt1 promoter. IRIF formed by mCherry-tagged Crb2(276–778) and Mdb1-GFP were imaged and quantified after exposure to 36 Gy of IR. Strains used were DY15912 and DY15602. Bar, 3 µm. (PDF) [file pone.0097028.s002.pdf]
