# Supplementary material for: Mdb1, a Fission Yeast Homolog of Human MDC1, Modulates DNA Damage Response and Mitotic Spindle Function
Source: PLoS One. 2014 May 7;9(5):e97028. doi: 10.1371/journal.pone.0097028 (PMC4013092; doi:10.1371/journal.pone.0097028)
Supplement: Table S1 — The strains used in this study. (PDF) [file pone.0097028.s003.pdf]

Table S1. Strains used in this study

Strain	Mating Type	Genotype
DY389	*h-*	*leu1-32 his3-D1 ura4+::Pmdb1-mdb1-YFP lys1-131::Pdis1-mCherry-LacI(lys1+) erg7ter::lacOrepeat(leu1+) arg3::HOsite-natMX(arg3-) ars1::pJR1-41XH+HO(his3+) rad52-2XCFP(kanMX) mdb1Δ::hphMX*
DY16261	*h+*	*ura4-D18 leu1-32 his3-D1 arg3::HOsite-natMX(arg3-) nmt1ter::pJR1-41XH+HO(his3+) rad52-2XFlag(kanMX) leu1-32::P41nmt1-GFP(leu1+)*
DY16263	*h+*	*ura4-D18 leu1-32 his3-D1 arg3::HOsite-natMX(arg3-) nmt1ter::pJR1-41XH+HO(his3+) rad52-2XFlag(kanMX) leu1-32::P41nmt1-Mdb1-wt-GFP(leu1+)*
DY15603	*h+*	*his3-D1 mdb1Δ::hphMX leu1-32::P81nmt1-Mdb1-wt-GFP(leu1+)*
DY15604	*h+*	*his3-D1 mdb1Δ::hphMX leu1-32::P81nmt1-Mdb1-S392A-GFP(leu1+)*
DY15605	*h+*	*his3-D1 mdb1Δ::hphMX leu1-32::P81nmt1-Mdb1-K434M-GFP(leu1+)*
DY15606	*h+*	*ura4-D18 his3-D1 mdb1Δ::hphMX hta1-S129A::ura4+ hta2-S128A::his3+ leu1-32::P81nmt1-Mdb1-wt-GFP(leu1+)*
DY15607	*h+*	*ura4-D18 his3-D1 mdb1Δ::hphMX set9Δ::kanMX leu1-32::P81nmt1-Mdb1-wt-GFP(leu1+)*
DY15631	*h+*	*ura4-D18 his3-D1 leu1-32::P81nmt1-Mdb1-wt-GFP(leu1+)*
DY15608	*h+*	*ura4-D18 ade6-M210 his3-D1 tel1Δ::kanMX leu1-32::P81nmt1-Mdb1-wt-GFP(leu1+)*
DY15609	*h-*	*ura4-D18 leu1-32 ade6-M210 his3-D1 rad3Δ::LEU2 ars1::P81nmt1-Mdb1-wt-GFP(ura4+)*
DY15610	*h?*	*ura4-D18 ade6-M210 his3-D1 tel1Δ::kanMX rad3Δ::LEU2 leu1-32::P81nmt1-Mdb1-wt-GFP(leu1+)*
LD327	*h-*	*his3-D1*
LD1067	*h-*	*leu1-32 his3-D1 mdb1Δ::natMX*
LD574	*h-*	*ura4-D18 leu1-32 his3-D1 hta1-S129A::ura4+ hta2-S128A::his3+*
LD197	*h-*	*ura4-D18 leu1-32 his3-D1 crb2Δ::ura4+*
LD1068	*h-*	*ura4-D18 leu1-32 his3-D1 mdb1Δ::natMX hta1-S129A::ura4+ hta2-S128A::his3+*
LD1070	*h-*	*ura4-D18 leu1-32 his3-D1 mdb1Δ::natMX crb2Δ::ura4+*

LD678	*h-*	*ura4-D18 leu1-32 his3-D1 hta1-S129A::ura4+ hta2-S128A::his3+ crb2Δ::ura4+*
LD1071	*h-*	*ura4-D18 leu1-32 his3-D1 mdb1Δ::natMX hta1-S129A::ura4+ hta2-S128A::his3+ crb2Δ::ura4+*
LD259	*h+*	*ura4-D18 leu1-32 his3-D1*
LD723	*h+*	*ura4-D18 leu1-32 his3-D1 set9Δ::kanMX*
LD964	*h+*	*ura4-D18 leu1-32 his3-D1 mdb1Δ::hphMX*
DY15611	*h+*	*ura4-D18 leu1-32 his3-D1 mdb1Δ::hphMX set9Δ::kanMX*
DY15612	*h+*	*ura4-D18 his3-D1 mdb1Δ::hphMX set9Δ::kanMX leu1-32::P81nmt1-GFP(leu1+)*
DY15613	*h+*	*ura4-D18 his3-D1 mdb1Δ::hphMX set9Δ::kanMX leu1-32::P81nmt1-Mdb1-S392A-GFP(leu1+)*
DY15614	*h+*	*ura4-D18 his3-D1 mdb1Δ::hphMX set9Δ::kanMX leu1-32::P81nmt1-Mdb1-K434M-GFP(leu1+)*
LD260	*h-*	*ura4-D18 leu1-32 his3-D1*
LD744	*h-*	*ura4-D18 leu1-32 crb2-F400A*
LD1011	*h-*	*ura4-D18 leu1-32 his3-D1 mdb1Δ::natMX*
DY16269	*h-*	*ura4-D18 leu1-32 his3-D1(or his3+) crb2-F400A mdb1Δ::natMX*
DY15615	*h+*	*ura4-D18 his3-D1 leu1-32::Pnmt1-GFP(leu1+)*
DY15616	*h+*	*ura4-D18 his3-D1 leu1-32::Pnmt1-Mdb1-wt-GFP(leu1+)*
DY15617	*h+*	*ura4-D18 his3-D1 leu1-32::Pnmt1-Mdb1-S392A-GFP(leu1+)*
DY15618	*h+*	*ura4-D18 his3-D1 leu1-32::Pnmt1-Mdb1-K434M-GFP(leu1+)*
DY15619	*h-*	*ura4-D18 his3-D1 set9Δ::kanMX leu1-32::Pnmt1-GFP(leu1+)*
DY15620	*h-*	*ura4-D18 his3-D1 set9Δ::kanMX leu1-32::Pnmt1-Mdb1-wt-GFP(leu1+)*
DY15621	*h-*	*ura4-D18 his3-D1 set9Δ::kanMX leu1-32::Pnmt1-Mdb1-S392A-GFP(leu1+)*
DY15622	*h-*	*ura4-D18 his3-D1 set9Δ::kanMX leu1-32::Pnmt1-Mdb1-K434M-GFP(leu1+)*
DY15623	*h+*	*ura4-D18 his3-D1 hta1-S129A::ura4+ hta2-S128A::his3+ leu1-32::Pnmt1-GFP(leu1+)*
DY15624	*h+*	*ura4-D18 his3-D1 hta1-S129A::ura4+ hta2-S128A::his3+ leu1-32::Pnmt1-Mdb1-wt-GFP(leu1+)*
DY15625	*h+*	*ura4-D18 his3-D1 hta1-S129A::ura4+ hta2-S128A::his3+ leu1-32::Pnmt1-Mdb1-S392A-GFP(leu1+)*
DY15626	*h+*	*ura4-D18 his3-D1 hta1-S129A::ura4+ hta2-S128A::his3+ leu1-32::Pnmt1-Mdb1-K434M-GFP(leu1+)*

DY15627	*h+*	*his3-D1 mdb1Δ::hphMX ars1::CFP-atb2::LEU2 leu1-32::P81nmt1-Mdb1-wt-GFP(leu1+)*
DY15628	*h+*	*his3-D1 mdb1Δ::hphMX ars1::CFP-atb2::LEU2 leu1-32::P81nmt1-Mdb1-S392A-GFP(leu1+)*
DY15629	*h+*	*his3-D1 mdb1Δ::hphMX ars1::CFP-atb2::LEU2 leu1-32::P81nmt1-Mdb1-K434M-GFP(leu1+)*
DY42	*h+*	*ura4-D18 leu1-32 his3-D1 ase1Δ::kanMX mdb1-YFP::natMX ars1::CFP-atb2::LEU2*
DY15630	*h+*	*leu1-32 his3-D1 mdb1Δ::hphMX leu1-32::P81nmt1-GFP(leu1+)*
DY15912	*h-*	*ura4-D18 his3-D1 mdb1Δ::hphMX leu1-32::P41nmt1-mCherry-crb2(276-778)(leu1+) ars1::Pnmt1-mdb1-wt-GFP(ura4+)*
DY15602	*h+*	*ura4-D18 his3-D1 mdb1Δ::hphMX leu1-32::P41nmt1-mCherry-crb2(276-778)(leu1+) ars1::P81nmt1-mdb1-wt-GFP(ura4+)*
