# Supplementary material for: Mdb1, a Fission Yeast Homolog of Human MDC1, Modulates DNA Damage Response and Mitotic Spindle Function
Source: PLoS One. 2014 May 7;9(5):e97028. doi: 10.1371/journal.pone.0097028 (PMC4013092; doi:10.1371/journal.pone.0097028)
Supplement: Table S2 — The PCR primers used in the ChIP assay. (PDF) [file pone.0097028.s004.pdf]

Table S2. PCR primers used in the ChIP assay

Name	Sequence	Description
HO-1-FP	CATAAGGTTTGCATACACCGTTGGGTAGG	3 kb from HO site
HO-1-RP	CGGAAAGAACTTGATTGGATTGATTAACACTC ATCC	3 kb from HO site
HO-2-FP	TAGCACCGGCTCGTCTATTT	5 kb from HO site
HO-2-RP	AAGCAATGGGACTTCAATCG	5 kb from HO site
HO-3-FP	TCAAAGCTGCGAAACAACAC	10 kb from HO site
HO-3-RP	TCGGTGCAGACGATCAATAA	10 kb from HO site
HO-4-FP	AGCAGGAGAGAGATATGGAACT	20 kb from HO site
HO-4-RP	GGGAAGTCTCCTACTTCAACTT	20 kb from HO site
HO-5-FP	GTATAGCGCCTGAAGAATTGAG	25 kb from HO site
HO-5-RP	ATTCGACCCGGGTTCGATTC	25 kb from HO site
act1-FP	TGCTCCTCCTGAGCGTAAATACTCTGTCTG	actin gene control
act1-RP	AACGATACCAGGTCCGCTCTCATCATACTC	actin gene control
nda2-FP	GCCGTTACTAGCATTAAATCTCGTCGCACC	tubulin gene control
nda2-RP	CTTAGCGATTCCACTACCAGGAACGTGTTG	tubulin gene control
